# Androgen‐targeting therapeutics mitigate the adverse effect of GnRH agonist on the risk of neurodegenerative disease in men treated for prostate cancer

**DOI:** 10.1002/cam4.4650

**Published:** 2022-03-16

**Authors:** Gregory L. Branigan, Georgina Torrandell‐Haro, Maira Soto, Edward P. Gelmann, Francesca Vitali, Kathleen E. Rodgers, Roberta Diaz Brinton

**Affiliations:** ^1^ Center for Innovation in Brain Science University of Arizona Tucson Arizona USA; ^2^ Department of Pharmacology University of Arizona College of Medicine Tucson Arizona USA; ^3^ Medical Scientist Training Program University of Arizona College of Medicine Tucson Arizona USA; ^4^ Department of Medicine, Division of Hematology and Oncology University of Arizona College of Medicine and University of Arizona Cancer Center Tucson Arizona USA; ^5^ Department of Neurology University of Arizona College of Medicine Tucson Arizona USA; ^6^ Center for Biomedical Informatics and Biostatistics University of Arizona Tucson Arizona USA

**Keywords:** abiraterone, Alzheimer's disease, androgen, medical informatics, neurodegenerative disease, prostate cancer

## Abstract

**Background:**

Prostate cancer and multiple neurodegenerative diseases (NDD) share an age‐associated pattern of onset. Therapy of prostate cancer is known to impact cognitive function. The objective of this study was to determine the impact of multiple classes of androgen‐targeting therapeutics (ATT) on the risk of NDD.

**Methods:**

A retrospective cohort study of men aged 45 and older with prostate within the US‐based Mariner claims data set between January 1 and 27, 2021. A propensity score approach was used to minimize measured and unmeasured selection bias. Disease risk was determined using Kaplan–Meier survival analyses.

**Results:**

Of the 1,798,648 men with prostate cancer, 209,722 met inclusion criteria. Mean (SD) follow‐up was 6.4 (1.8) years. In the propensity score‐matched population, exposure to ATT was associated with a minimal increase in NDD incidence (relative risk [RR], 1.07; 95% CI, 1.05–1.10; *p* < 0.001). However, GnRH agonists alone were associated with significantly increased NDD risk (RR, 1.47; 95% CI, 1.30–1.66; *p* <0.001). Abiraterone, commonly administered with GnRH agonists and low‐dose prednisone, was associated with a significantly decreased risk (RR, 0.77; 95% CI, 0.68–0.87; *p* < 0.001) of any NDD.

**Conclusions:**

Among patients with prostate cancer, GnRH agonist exposure was associated with an increased NDD risk. Abiraterone acetate reduced the risks of Alzheimer's disease and Parkinson's disease conferred by GnRH agonists, whereas the risk for ALS was reduced by androgen receptor inhibitors. Outcomes of these analyses contribute to addressing controversies in the field and indicate that GnRH agonism may be a predictable instigator of risk for NDD with opportunities for risk mitigation in combination with another ATT.

## Graphical abstract

Assessment of long‐term risk of developing neurodegenerative diseases in a clinical population of men with prostate cancer treated with androgen‐targeting therapeutics using medical bioinformatics tools. This figure was created with BioRender.com.

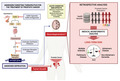

## 1 INTRODUCTION

Prostate cancer is the most prevalent non‐skin cancer in men and the second leading cause of cancer‐related deaths among men worldwide,^1^ accounting for 29% of new cancer cases.^2^ In 2022, 268,490 new cases of prostate cancer are expected to be diagnosed in the U.S..^2^ Androgen deprivation therapy has been effectively used for the treatment of prostate cancer for more than 75 years.^3^, ^4^, ^5^, ^6^, ^7^, ^8^

Gonadotrophin‐releasing hormone (GnRH) agonists (leuprolide, goserelin, triptorelin, and histrelin) and GnRH antagonists (degarelix) constitute the drug class of androgen deprivation therapy (ADT) in the U.S.^3^, ^8^, ^9^ Recently, androgen synthesis inhibitors (ketoconazole and abiraterone)^10^, ^11^ or androgen receptor (AR) inhibitors (flutamide, bicalutamide, nilutamide, and enzalutamide)^12^, ^13^ were added to improve the efficacy of GnRH agonists and antagonists.^14^ Although abiraterone's mechanism of action is different from direct AR blockade, the clinical benefits have been indistinguishable from third‐generation AR antagonists despite the absence of direct comparisons in clinical trials.^15^, ^16^, ^17^, ^18^ Both androgen receptor inhibitors and androgen synthesis inhibitors are administered in combination with GnRH agonists, antagonists, or orchiectomy, consistent with the results of randomized clinical trials and FDA approvals for these agents.

There are well‐documented neurocognitive side effects due to androgen ablation that have raised concerns regarding a potential association between androgen deprivation and neurodegenerative disease.^19^, ^20^, ^21^, ^22^, ^23^, ^24^, ^25^, ^26^, ^27^, ^28^, ^29^, ^30^, ^31^, ^32^, ^33^, ^34^, ^35^ Reduced plasma level of testosterone has been reported in Alzheimer's, Multiple sclerosis, Parkinson's, and ALS (reviewed in Bianchi et al., 2020).^36^ In Alzheimer's disease, reduced circulating and brain testosterone was reported and correlated with AD pathology suggesting that low testosterone is a factor that contributes to, rather than results from, the development of AD (reviewed in Rosario et al., 2008).^37^, ^38^

Despite the strong associations with decreased testosterone levels and neurodegenerative disease, the impact of ATT and risk of neurodegenerative diseases,^39^ including Alzheimer's disease (AD)^21^, ^30^ Multiple sclerosis (MS),^40^ Parkinson's disease (PD),^41^, ^42^ and Amyotrophic Lateral Sclerosis (ALS),^43^, ^44^ remain controversial with few studies reporting the association with androgen receptor and synthesis inhibitors. To address these issues, we conducted analyses of the relationship between androgen‐targeting therapeutics with different mechanisms of action and the incidence of age‐related neurodegenerative diseases.

## 2 METHODS

### 2.1 Data source

The Mariner database is an insurance claims data set that serves the United States with patient populations from all US states and territories. The Mariner data set contains patient demographic characteristics, prescription records, and numerous other data points for patients with *Current Procedural Terminology*, *International Classification of Diseases, Ninth Revision (ICD‐9)*, and *International Statistical Classification of Diseases and Related Health Problems, Tenth Revision (ICD‐10)* codes. As of October 2020, Mariner encompassed all indications and represents 122 million patients throughout the duration of the set with claims dating from 2010 through the second quarter of 2018.

This report follows the Strengthening the Reporting of Observational Studies in Epidemiology (STROBE) reporting guideline. This study was approved by the University of Arizona Institutional Review Board. Requirements for informed consent were waived as the data were deidentified.

### 2.2 Study design and variables

**FIGURE 1:**
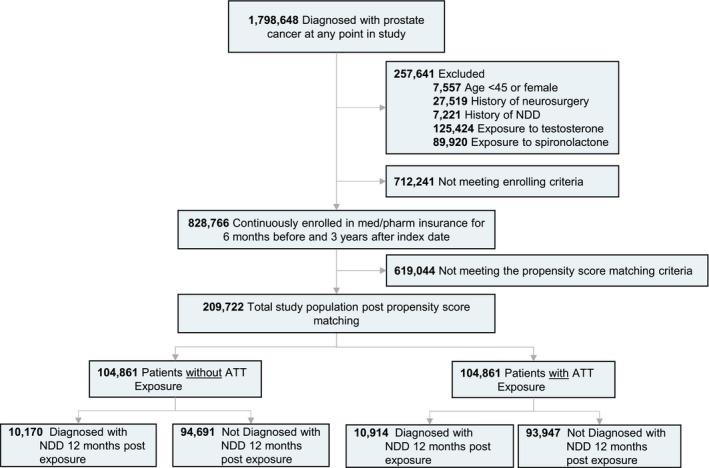
Study design and patient breakdown. NDD, Neurodegenerative Diseases; ATT, Androgen‐Targeting Therapeutics

The subset of 1,798,648 prostate cancer patients was generated from the available Mariner data set for the study group. Participants younger than 45 years old, with a history of neurosurgery, brain cancer, or neurodegenerative disease prior to the diagnosis of prostate cancer, history of exposure to spironolactone or testosterone were excluded from the study. A 3‐year active enrollment criterion after the diagnosis of prostate cancer was required before analysis of exposure to ATT or development of NDD for all patients (Figure 1) to account for patients that may be leaving, dying, or changing insurance providers. Additionally, the 3‐year follow‐up is not based on ATT exposure but instead is based on prostate cancer diagnosis. Patient groups were assigned according to therapeutic intervention used secondary to a prostate cancer diagnosis after meeting enrollment criteria to avoid immortal bias in experimental design. The ATT group was defined as any patient having at least one medication charge for any of the ATT therapeutics occurring after the diagnosis of prostate cancer (Table S1). The outcome variable was defined as the occurrence of the first diagnosis of neurodegenerative disease based on ICD‐9 and ICD‐10 codes in the patient's medical claims data starting 1 year after a prostate cancer diagnosis. We defined neurodegenerative disease to include Alzheimer's disease (AD), non‐AD dementia, Multiple Sclerosis (MS), Parkinson's disease (PD), and Amyotrophic Lateral Sclerosis (ALS) (Table S1). An index date 1 year after the diagnosis of prostate cancer was selected to eliminate any acute neurocognitive side effects and to focus on the long‐term impact on disease progression given the prodromal nature of NDD. Age in the study is defined by the age at diagnosis of prostate cancer. Following the analytic strategy in Branigan et al. (2020)^45^ and Torrandell‐Haro et al. (2020),^46^ an analysis of comorbidities known to be associated with NDD outcomes was conducted (Table 1, Table S1).

**TABLE 1:** Baseline characteristics for unadjusted and propensity score‐matched patients with or without exposure to androgen‐targeting therapeutics

	Unadjusted cohort						Propensity score‐matched cohort^a^					
	Without exposure to ATT		With exposure to ATT				Without exposure to ATT		With exposure to ATT			
	*n*	%	*n*	%			*n*	%	*n*	%		
Number of Patients	723,905		104,861		*p* value	Std Mean Diff	104,861		104,861		*p* value	Std Mean Diff
Age												
45–49	36,098	4.99%	4273	4.07%	<0.001	0.503	4243	4.05%	4273	4.07%	<0.001	0.256
50–54	85,946	11.87%	9386	8.95%			10,411	9.93%	9386	8.95%		
55–59	106,607	14.73%	12,803	12.21%			14,328	13.66%	12,803	12.21%		
60–64	123,150	17.01%	16,256	15.50%			17,719	16.90%	16,256	15.50%		
65–69	123,498	17.06%	19,073	18.19%			18,483	17.63%	19,073	18.19%		
70–74	187,422	25.89%	32,174	30.68%			30,336	28.93%	32,174	30.68%		
75–79	61,184	8.45%	10,896	10.39%			9341	8.91%	10,896	10.39%		
Region												
Midwest	181,296	25.04%	21,558	20.56%	<0.001	0.186	26,123	24.91%	21,558	20.56%	<0.001	0.038
Northeast	169,690	23.44%	25,938	24.74%			24,849	23.70%	25,938	24.74%		
South	280,049	38.69%	43,787	41.76%			39,241	37.42%	43,787	41.76%		
West	92,104	12.72%	13,463	12.84%			14,548	13.87%	13,463	12.84%		
Unknown	766	0.11%	115	0.11%			100	0.10%	115	0.11%		
Comorbidities												
Asthma	6739	0.93%	2271	2.17%	<0.001	0.038	1169	1.11%	1253	1.19%	0.09	0.012
COPD	13,266	1.83%	2912	2.78%	<0.001	0.032	2091	1.99%	2388	2.28%	<0.001	0.006
Chronic Kidney Disease	30,565	4.22%	8104	7.73%	<0.001	0.066	4862	4.64%	5995	5.72%	<0.001	0.023
Congestive Heart Failure	20,403	2.82%	4609	4.40%	<0.001	0.025	3163	3.02%	3785	3.61%	<0.001	0.018
Coronary Artery Disease	54,876	7.58%	13,316	12.70%	<0.001	0.070	8916	8.50%	10,029	9.56%	<0.001	0.013
Ischemic Heart Disease	51,538	7.12%	11,832	11.28%	<0.001	0.062	8381	7.99%	9461	9.02%	<0.001	0.010
Obesity	36,611	5.06%	9475	9.04%	<0.001	0.059	5863	5.59%	6883	6.56%	<0.001	0.018
Osteoarthritis	61,832	8.54%	14,576	13.90%	<0.001	0.078	10,189	9.72%	11,475	10.94%	<0.001	0.011
Pulmonary Heart Disease	10,785	1.49%	2911	2.78%	<0.001	0.089	1727	1.65%	2071	1.97%	<0.001	0.012
Rheumatoid Arthritis	4149	0.57%	1103	1.05%	<0.001	0.028	686	0.65%	867	0.83%	<0.001	0.010
Stroke	28,366	3.92%	6743	6.43%	<0.001	0.059	4672	4.46%	5260	5.02%	<0.001	0.008
CCI												
0	328,635	45.40%	41,828	39.89%	0.39	0.724	42,322	40.36%	41,828	39.89%	>0.99	0.223
1	154,810	21.39%	21,206	20.22%			22,085	21.06%	21,206	20.22%		
2	109,748	15.16%	19,152	18.26%			17,872	17.04%	19,152	18.26%		
3	58,257	8.05%	9858	9.40%			9794	9.34%	9858	9.40%		
4	27,794	3.84%	4736	4.52%			4699	4.48%	4736	4.52%		
5–10	41,573	5.74%	7559	7.21%			7452	7.11%	7559	7.21%		
11+	3088	0.43%	522	0.50%			637	0.61%	522	0.50%		

Abbreviations: ATT, Androgen‐Targeting Therapeutics; COPD, Chronic Obstructive Pulmonary Disease; CCI, Charlson Comorbidity Index.

^a^ Adjusted for age, region, comorbidities, and CCI.

### 2.3 Statistical analysis

Statistical analyses were conducted between January 1 and January 27, 2021. Patient demographic statistics (Table 1) and incidence statistics were analyzed using unpaired two‐tailed *t* tests or χ2 tests, as appropriate, to test the significance of the differences between continuous and categorical variables. In all analyses, a two‐sided *p* <0.01 was considered statistically significant.

A propensity score‐matched population was generated by using logistic regression to identify confounding factors for ATT usage as outcome between the treatment and control groups as previously reported.^45^ In brief, the resulting factors included age, region, Charlson Comorbidity Index (CCI) rank as well as variable comorbidities listed in Table S1 which special attention is given to factors associated with NDD risk. CCI was calculated at the time of prostate cancer diagnosis. These factors were then integrated to match patients using a 1:1 ratio in the treatment (ATT) group to patients in the control (no‐ATT exposure) group to minimize confounding variables in the patient populations. The matching was assessed by standardized mean difference with percentage balance improvement (Table S2). Kaplan–Meier survival curves for NDD‐Free Survival were created using the propensity score‐matched population in the Bellwether–PearlDiver interface. We then conducted a sensitivity analysis to address the impact of surgery (orchiectomy or prostatectomy) and prednisone in the study population (Table S3; Table S4; Figure S4).

Biological pathway analysis was conducted using the Drug–Target Interaction (DTI) network approach (Figure S3). For each ATT identified, the related gene targets were extracted using the DrugBank database.^47^

## 3 RESULTS

Of the 1,798,648 prostate cancer patients within the Mariner data set, 828,766 met the inclusion/exclusion criteria and claims enrollment period requirements for analysis (Figure 1). After 1:1 propensity score matching, 209,722 patients remained in the study (Figure 1). In the postmatching group, 104,861 patients (mean [SD] age, 62.83 [3.56] years) were assigned to the control group and 104,861 patients (mean [SD] age, 63.42 [3.77] years) were assigned to the ATT treatment group. ATT was started on average (SD) 1114.36 (822.62) days after the diagnosis of prostate cancer, consistent with the expectation that most men initiated ATT for either biochemical progression or the discovery of the metastatic disease. The mean (interquartile range) filled prescription days was 2326 (IQR, 1772–2915). Drugs defined as ATT, patient counts, and median adherence rate for each drug are reported in Table 2. Generic drug codes used within the PearlDiver database are included in Table S2. Patient groups were then followed for the duration of their claims data entries and surveyed for any diagnosis of NDD. The mean (SD) follow‐up was 6.4 (1.8) years.

**TABLE 2:** List of androgen‐targeting therapeutics and number of patients per group

Group	Drug	*n* (%)	Median % adherence
GnRH agonists	Goserelin	67 (0.06)	81.62
	Histrelin	15 (0.01)	80.89
	Leuprolide	1216 (1.08)	—
	Triptorelin	352 (0.31)	85.91
GnRH antagonists	Degarelix	117 (0.10)	90.81
Androgen receptor inhibitors	Bicalutamide	17,949 (15.96)	85.23
	Enzalutamide	2549 (2.27)	—
	Flutamide	426 (0.38)	80.54
	Nilutamide	166 (0.15)	94.49
Androgen synthesis inhibitors	Abiraterone	2993 (2.66)	92.98
	Ketoconazole	86,638 (77.02)	16.44

In the unadjusted cohort, age, region, comorbidity rates indicated statistically significant differences between the control and ATT exposure group (Table 1). Most patients were from the southern region of the United States (280,049 of 723,905 [38.69%] and 43,787 of 104,861 [41.76%]). The age range of the study population was 45–79 for both the control and ATT‐exposed groups, where the majority of patients were aged 70–74 (187,422 controls of 723,905 [25.89%] and 32,174 ATT treated of 104,861 [30.68%]). Incidence of 11 representative comorbidities associated with NDD that were different between the control and treatment groups are reported in Table 1. CCI was not statistically different between the unadjusted control and the ATT treatment group. To address the impact of the above demographic differences between groups, propensity score matching was conducted to generate representative groups that controlled for differences in demographic and comorbid characteristics. Demographics of populations generated by propensity matching appear in Table 1.

In the unadjusted population, exposure to ATT was associated with an increased risk of all combined neurodegenerative diseases (NDD) (65,278 of 723,905 [9.02%] vs. 11,127 of 104,861 [10.61%]; relative risk [RR], 1.18; 95% CI, 1.16–1.20; *p* < 0.001) as well as AD, non‐AD Dementia, MS, and PD. There was no statistical difference in ATT exposure to ALS risk in the overall ATT unadjusted population (Table 3). In the propensity score‐adjusted population, statistical differences were sustained for NDD (10,170 of 104,861 [9.70%] vs. 10,914 of 104,861 [10.41%]; relative risk [RR], 1.07; 95% CI, 1.05–1.10; *p* < 0.001), and PD (2586 of 104,861 [2.47%] vs. 3534 of 104,861 [3.37%]; relative risk [RR], 1.37; 95% CI, 1.30–1.44; *p* < 0.001). In contrast, the impact of the ATT group was no longer statistically significant for AD (2939 of 104,861 [2.80%] vs. 3025 of 104,861 [2.88%]; relative risk [RR], 1.03; 95% CI, 0.98–1.08; *p* = 0.26), non‐AD dementia (5069 of 104,861 [4.83%] vs. 5159 of 104,861 [4.92%]; relative risk [RR], 1.02; 95% CI, 0.98–1.06; *p* = 0.37), and MS (420 of 104,861 [0.40%] vs. 416 of 104,861 [0.40%]; relative risk [RR], 0.99; 95% CI, 0.87–1.13; *p* = 0.92) postmatching in the ATT exposure group. Propensity score–matched data are presented in Figure S1. Kaplan–Meier survival curves for NDD‐free survival for each NDD subtype were generated using propensity score–matched population data to evaluate incidence rate and percent of population for each disease (Figure S2). Observed changes in the rate of disease incidence between control patient and those patients receiving ATT confirmed the results shown in Table 3.

**TABLE 3:** Relative risk of unadjusted and propensity score‐matched patients with or without exposure to ATT to develop NDDs

	All NDD combined	AD	Non‐AD dementia	MS	PD	ALS
Unadjusted cohort						
Patients not receiving Androgen‐Targeting Therapy	65,278	18,975	32,428	2431	17,060	618
%	9.02%	2.62%	4.48%	0.34%	2.36%	0.09%
Patients receiving Androgen‐Targeting Therapy	11,127	3096	5288	432	3600	85
%	10.61%	2.95%	5.04%	0.41%	3.43%	0.08%
Relative risk	1.18	1.13	1.13	1.23	1.46	0.95
95% CI	1.16–1.20	1.09–1.17	1.09–1.16	1.11–1.36	1.41–1.51	0.76–1.19
NNT	62.75	301.9	177.5	1313	92.9	23,198
*p* value	<0.001	<0.001	<0.001	<0.001	<0.001	0.69
Propensity score‐matched cohort						
Patients not receiving Androgen‐Targeting Therapy	10,170	2939	5069	420	2586	86
%	9.70%	2.80%	4.83%	0.40%	2.47%	0.08%
Patients receiving Androgen‐Targeting Therapy	10,914	3025	5159	416	3534	83
%	10.41%	2.88%	4.92%	0.40%	3.37%	0.08%
Relative risk	1.07	1.03	1.02	0.99	1.37	0.97
95% CI	1.05–1.10	0.98–1.08	0.98–1.06	0.87–1.13	1.30–1.44	0.71–1.30
NNT	140.9	1219	1165	26,215	110.6	34,954
*p* value	<0.001	0.26	0.37	0.92	<0.001	0.88

Abbreviations: ATT, Androgen‐Targeting Therapeutics; NDD, Neurodegenerative Diseases; AD, Alzheimer's Disease; MS, Multiple Sclerosis; PD, Parkinson's Disease; ALS, Amyotrophic Lateral Sclerosis; CI, Confidence Interval; NNT, Number Needed to Treat.

To assess the strength of the association between ATT and diagnosis of NDD, multiple types of sensitivity analyses were conducted. Subgroups of treatment combinations were analyzed including surgical interventions of prostatectomy and orchiectomy (Table S3). In patients without surgical intervention, ATT use had a higher hazard of diagnosis for all neurodegenerative disease (RR, 1.48; 95% CI, 1.44–1.53, *p* value < 0.001). In patients with surgery, only the orchiectomy population was associated with an increased risk of neurodegenerative diseases (RR, 2.25; 95% CI, 1.65–3.03, *p* value < 0.001) further strengthening the validity of the association as prostatectomy could be considered a negative surgical control (Table S3). Further, because abiraterone is almost always prescribed with low doses of prednisone, consistent with the FDA approval, a subanalysis was conducted to (1) address clinical prescribing guidelines and (2) determine if prednisone exposure was associated with impact on NDD in this drug type (Table S4). Consistent with medical practice, the majority of patients (90.6%) received prednisone with abiraterone. Outcomes of subanalysis indicated no association between prednisone and NDD providing support that the reduced NDD risk reduction profile was driven by abiraterone exposure (Table S4).

**FIGURE 2:**
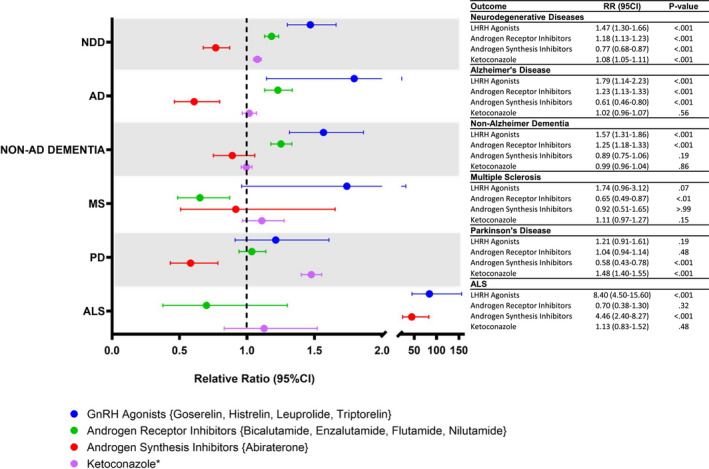
Relative risk of propensity score‐matched prostate cancer patients with exposure to different ATT classes to develop NDD. *Ketoconazole is considered an Androgen Synthesis Inhibitor. ATT, Androgen‐Targeting Therapeutics; NDD, Neurodegenerative Diseases; AD, Alzheimer's Disease; MS, Multiple Sclerosis; PD, Parkinson's Disease; ALS, Amyotrophic Lateral Sclerosis; RR, Relative Risk; CI, Confidence Interval; GnRH. Luteinizing Hormone‐Releasing Hormone

Further, an analysis of the incidence of each NDD with exposure to a subclass of ATT was conducted to address the selectivity of ATT drug action. Patients receiving ATT were divided into four groups based on therapeutic mechanism: GnRH agonists, androgen receptor inhibitors, androgen synthesis inhibitors, and ketoconazole, a drug used prior to the introduction of abiraterone due to its low‐affinity inhibition of androgen synthesis (Figure 2). Of note, androgen receptor inhibitors and androgen synthesis inhibitors are prescribed in combination with GnRH agonists or antagonists, thus, the two groups in this analysis represent a combined therapeutic exposure. GnRH antagonists were not included in this analysis as there were too few patients in the cohort receiving these therapies. Mechanistic pathway targets of these drugs are presented in Figure S4.

The outcomes of these analyses indicated that in contrast to the analysis of all ATT as an overall group, subgroups of ATT therapies were associated with distinct and statistically significant risk profiles for each NDD subtype. GnRH agonists and androgen receptor inhibitors were both associated with an increased risk of AD (RR, 1.79; 95% CI, 1.14–2.23; and RR, 1.23; 95% CI, 1.13–1.33; *p* <0.001), non‐AD dementia (RR, 1.57; 95% CI, 1.31–1.86; and RR, 1.25; 95% CI, 1.18–1.33; *p* <0.001). These subgroups represent GnRH agonists and a given second‐line ATT. In contrast to the increased risk profile of GnRH agonists alone, the addition of abiraterone was associated with a significantly reduced risk of AD (RR, 0.61; 95% CI, 0.46–0.80; *p* < 0.001) and a non‐significant effect on non‐AD dementia (RR, 0.89; 95% CI, 0.75–1.06; *p* = 0.29). Of note, ketoconazole did not show a statistically significant impact on AD or dementia risk. For MS, the administration of androgen receptor inhibitors was associated with a decreased risk (RR, 0.65; 95% CI, 0.49–0.87, *p* < 0.01), whereas the other therapies did not significantly impact the risk of MS. For PD, abiraterone was associated with a decreased risk (RR, 0.58; 95% CI, 0.43–0.78; *p* < 0.001), whereas ketoconazole was associated with an increased risk (RR, 1.48; 95% CI, 1.40–1.55; *p* < 0.001). For ALS, abiraterone (RR, 4.46; 95% CI, 2.40–8.27; *p* < 0.001) and androgen receptor inhibitors (RR, 8.40; 95% CI, 4.50–15.60; *p* < 0.001) were associated with a significantly increased risk of ALS (Figure 2).

**FIGURE 3:**
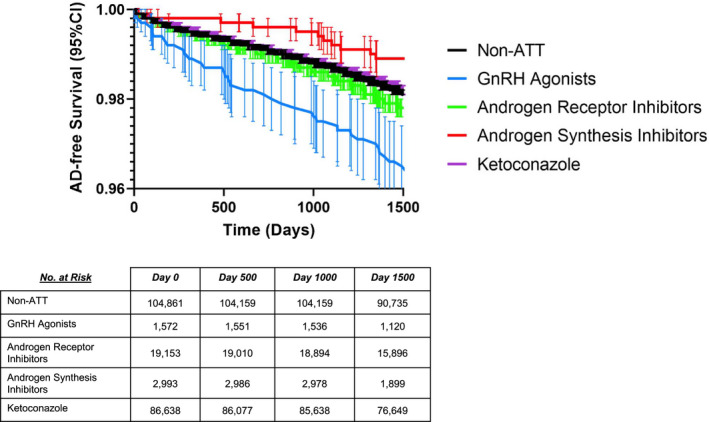
Kaplan–Meier AD survival curves for propensity score‐matched prostate cancer patients with exposure to different ATT classes. ATT, Androgen‐Targeting Therapeutics; AD, Alzheimer's Disease; CI, Confidence Interval; GnRH. Luteinizing Hormone‐Releasing Hormone

To further evaluate the impact of individual ATT drug classes on NDD risk, survival analysis was conducted for AD in populations stratified by the ATT group (Figure 3). Kaplan–Meier probability analyses confirmed the associations of chi‐square analyses and illustrated that GnRH agonists alone exerted the greatest acceleration of AD incidence, followed by androgen receptor inhibitors, and ketoconazole.

## 4 DISCUSSION

The analyses described herein are the largest report to date examining the impact of a broad range of androgen‐targeting therapies that included androgen receptor inhibitors and androgen synthesis inhibitors on neurodegenerative disease incidence in a cohort of men with prostate cancer. Previous studies focused on the androgen deprivation therapy (ADT) drug class in reporting associations with AD and dementia risk.^21^, ^24^, ^27^, ^31^ These studies reported variable results with controversies around controls and confounding factors.^25^, ^48^, ^49^, ^50^ A recent meta‐analysis evaluating the association between androgen deprivation therapy and dementia or Alzheimer's disease (AD) found a positive association.^21^ However, the study was limited to the effect of GnRH agonists and antagonists. Consistent with clinical practice, our study determined the impact of ATT drug combination risk profiles for adjuvant ATT to GnRH agonists. Further, we extended analyses that determined the impact of ATT on a broad range of NDD disorders including non‐AD dementia, Parkinson's, MS, and ALS. Outcomes of these analyses reported herein will be useful as a comparison to analyses of a single class of ATT which may provide insights into variances across reports which may be, in part, due to the therapies selected and, in part, due to the control group used for comparison (healthy control vs prostate cancer patients without ATT exposure).^25^, ^28^, ^29^, ^31^, ^51^, ^52^, ^53^

When taken together as a group, the impact of ATT on all NDD is associated with a slight increase in NDD incidence which was driven by the incidence of Parkinson's disease, whereas the remaining disease incidences were not statistically significantly different from the control (Figure S1). When stratified into subgroup drug classes (e.g., synthesis or receptor inhibitors), the results indicated three risk profiles associated with exposure to a given class of ATT. For AD and non‐AD dementia, GnRH agonists and androgen receptor inhibitors were associated with an increased risk. Conversely, exposure to the second‐generation androgen synthesis inhibitor, abiraterone, was associated with decreased incidence of AD and non‐AD dementia (Figure 2). Of note, exposure to ketoconazole, which had been used as an androgen synthesis inhibitor, had no impact on the risk of AD or non‐AD dementia. Based on the drug‐target network generated from the biological pathway analysis, ketoconazole targets both the androgen receptor and CYP21A2 (21‐Hydroxylase) among others (Figure S3) but is known to be less potent than abiraterone in affecting androgenesis and less specific.^15^, ^16^, ^51^, ^52^, ^53^ Moreover, the biological pathway analysis indicated that flutamide targets NR1I2 and AHR in addition to the androgen receptor and that goserelin targets LHCGR in addition to GNRHR. Further research is needed to evaluate if these differential targets exert an impact on the risk of NDD associated with these drug groups.

Similar risk profiles for individual ATT exposure occurred for MS, PD, and ALS with variances in which therapeutics were driving the protective or increased risk profiles. As noted for MS, exposure to the androgen receptor inhibitors was associated with a decreased risk, whereas for PD exposure to ketoconazole was associated with an increased risk. Most notably, both GnRH agonists and abiraterone were associated with a significantly increased risk of ALS (Figure 2 **).**

The mechanisms underlying the relationship between androgen loss and NDD are likely complex and involve multiple systems of biology. Mechanistically, the different risk profiles associated for each of the NDD may be due, in part, to general or specific inhibition and subsequent feedback within the androgen pathway. ATT represents a heterogeneous group of medications with distinct mechanisms and physiologic effects. For example, GnRH agonists cause an initial surge in testosterone via luteinizing hormone (LH), whereas therapeutics that block androgen receptors, no surge is experienced.^49^, ^54^, ^55^ Additionally, each therapeutic class has different effects on follicle‐stimulating hormone and LH, which in turn, impact the inflammatory, vascular, and metabolic systems.^56^, ^57^ Thus, it follows that there can be differential risk profiles and neurological adverse events between forms of ATT. The data reported herein indicate that mechanisms of action of ATT can differentially impact outcomes on the risk of age‐associated neurodegenerative diseases and illustrates the complexity of interaction between androgen pathways and the nervous system.

## 5 LIMITATIONS

As a retrospective analysis of a claims database, there are important limitations to consider. Patients included may have obtained services outside those included in this database and there could be factors, known and unknown, that even with propensity matching may not be adequately addressed. Claim records do not include the results of cognitive exams or lab results thus a diagnosis of the disease is based on the presence of ICD codes associated with a given disease which may represent a source of inaccuracy. In addition, data on specific prostate pathologic conditions on contraindications for therapy cannot be assessed in this data set. With respect to patient profiles, small differences in comorbidity may reflect the different disease state of exposed and unexposed patients, which we are unable to fully account for with the propensity score matching. In this respect, this data set does not include lab or genetic values nor pathological staging and was not available for inclusion in the analyses. These limitations are reported here for reference compared with clinical trial data. Despite these limitations, the outcomes of analyses reported herein represent clinically relevant observational findings regarding the association of ATT exposure and NDD risk.

## 6 CONCLUSIONS

Outcomes of these analyses contribute to addressing controversies in the field and indicate that GnRH agonism to induce cessation of gonadal steroid production is associated with the greatest risk for NDD. However, combination ATT therapy indicated potential risk mitigation with targeted androgen synthesis inhibition exhibiting the greatest risk reduction. Further investigation of the direct and combinatorial/adjuvant effects of androgen‐targeting therapeutics on diseases of the nervous system is warranted and could improve patient compliance given the growing concern of neurological impairment in aging populations.^58^

## CONFLICT OF INTEREST

Dr Brinton reported receiving grants from the National Institute on Aging and the Women's Alzheimer's Movement during the conduct of the study. GLB, GTH, MS, KER, EPG, and RDB have submitted a patent for the use of abiraterone to mitigate the risk of neurodegenerative disease. No other disclosures were reported.

## AUTHOR CONTRIBUTIONS

Mr. Branigan, Miss Torrandell‐Haro, Dr. Kathleen Rodgers, and Dr. Roberta Brinton had full access to all the data in the study and took responsibility for the integrity of the data and the accuracy of the data analysis. Mr. Branigan and Miss Torrandell‐Haro are co‐first authors. Concept and design: All authors. Acquisition, analysis, or interpretation of data: All authors. Drafting of the manuscript: All authors. Critical revision of the manuscript for important intellectual content: All authors. Statistical analysis: Torrandell, Branigan. Obtained funding: Brinton. Administrative, technical, or material support: Brinton, Rodgers. Supervision: Brinton.

## Supporting information


Table S1
Table S2
Table S3
Table S4
Figure S1
Figure S2
Figure S3
Figure S4

## Data Availability

The data that support the findings of this study are available from PearlDiver Inc. Restrictions apply to the availability of these data, which were used under license for this study. Data are available from the authors with the permission of PearlDiver Inc.
