# Supplementary material for: Androgen‐targeting therapeutics mitigate the adverse effect of GnRH agonist on the risk of neurodegenerative disease in men treated for prostate cancer
Source: Cancer Med. 2022 Mar 16;11(13):2687–98. doi: 10.1002/cam4.4650 (PMC9249980; doi:10.1002/cam4.4650)
Supplement: Supplementary file 1 — Table S1 Table S2 Table S3 Table S4 Figure S1 Figure S2 Figure S3 Figure S4 [file CAM4-11-2687-s001.pdf]

eTable 1. List of ICD, DRUG, and CPT diagnosis codes used in analysis

eTable1. List of Diagnose

Diagnosis	ICD-9	ICD-10	DRUG CODE	CTP
Alzheimer's Disease	ICD-9-D-3310	ICD-10-D-G300, ICD-10-D-G301, ICD-10-D-G308, ICD-10-D-G309		
Acute Myocardial Infarction	ICD-9-D-410:ICD-9-D-41099, ICD-9-D-412:ICD-9-D-41299	ICD-10-D-I21:ICD-10-D-I2199, ICD-10-D-I22:ICD-10-D-I2299, ICD-10-D-I252		
Alcohol Abuse	ICD-9-D-2652, ICD-9-D-2911:ICD-9-D-2913, ICD-9-D-2915:ICD-9-D-2919, ICD-9-D-3030:ICD-9-D-3039, ICD-9-D-30500:ICD-9-D-30503, ICD-9-D-3575, ICD-9-D-4255, ICD-9-D-53530, ICD-9-D-53531, ICD-9-D-5710:ICD-9-D-5713, ICD-9-D-9800:ICD-9-D-9809, ICD-9-D-V113	ICD-10-D-F101:ICD-10-D-F1099, ICD-10-D-E52, ICD-10-D-G621, ICD-10-D-I426, ICD-10-D-K2920, ICD-10-D0K2921, ICD-10-D-K700, ICD-10-D-K7030, ICD-10-D-K7031, ICD-10-D-K709, ICD-10-D-T510:ICD-10-D-T5195, ICD-10-D-Z502, ICD-10-D-Z7141, ICD-10-D-Z7142, ICD-10-D-Z721		
ALS	ICD-9-D-33520	ICD-10-D-G1221		
Asthma	ICD-9-D-49300, ICD-9-D-49399	ICD-10-D-J452:ICD-10-D-J45988		
Blood Loss Anemia	ICD-9-D-2800	ICD-10-D-D500		
COPD	ICD-9-D-490:ICD-9-D-49699	ICD-10-D-J44:ICD-10-D-J449		
Cancer	ICD-9-D-140:ICD-9-D-17299, ICD-9-D-174:ICD-9-D-195899, ICD-9-D-200:ICD-9-D-20899, ICD-9-D-2386, ICD-9-D-1400:ICD-9-D-1729, ICD-9-D-1740:ICD-9-D-1959	ICD-10-D-C00:ICD-10-D-C2699, ICD-10-D-C30:ICD-10-D-C3499, ICD-10-D-C37:ICD-10-D-C4199, ICD-10-D-C43:ICD-10-D-C4399, ICD-10-D-C45:ICD-10-D-C5899, ICD-10-D-C60:ICD-10-D-C7699, ICD-10-D-C81:ICD-10-D-C8599, ICD-10-D-C88:ICD-10-D-C8899, ICD-10-D-CC90:ICD-10-D-C9799, ICD-10-D-C000:ICD-10-D-C269, ICD-10-D-C300:ICD-10-D-C3492, ICD-10-D-C370:ICD-10-D-C419, ICD-10-D-C430:ICD-10-D-C439, ICD-10-D-C450:ICD-10-D-C58, ICD-10-D-C600:ICD-10-D-C768, ICD-10-D-C970:ICD-10-D-C979		
Cardiac Arrhythmias	ICD-9-D-4260, ICD-9-D-42613, ICD-9-D-4267, ICD-9-D-4269, ICD-9-D-42610, ICD-9-D-42612, ICD-9-D-4270:ICD-9-D-4274, ICD-9-D-4276ICD-9-D-4279, ICD-9-D-7850, ICD-9-D-99601, ICD-9-D-99604, ICD-9-D-V450, ICD-9-D-V533	ICD-10-D-I441:ICD-10-D-I4435, ICD-10-D-I456, ICD-10-D-I459, ICD-10-D-I470:ICD-10-D-I499, ICD-10-D-ROOO, ICD-10-D-ROO1, ICD-10-D-ROO8, ICD-10-D-T82100:ICD-10-D-T82199S, ICD-10-D-Z4501:ICD-10-D-Z4509, ICD-10-D-Z950		
Cerebrovascular Disease	ICD-9-D-36234, ICD-9-D-430:ICD-9-D-43899	ICD-10-D-G45:ICD-10-D-G4599, ICD-10-D-G46:ICD-10-D-G4699, ICD-10-D-H340, ICD-10-D-I60:ICD-10-D-I6999		
Chronic Kidney Disease	ICD-9-D-585, ICD-9-D-5851, ICD-9-D-5852, ICD-9-D-5853, ICD-9-D-5854, ICD-9-D-5855, ICD-9-D-5856, ICD-9-D-5859, ICD-9-D-7925	ICD-10-D-N18:ICD-10-D-N189		
Chronic Pulmonary Disease	ICD-9-D-4168, ICD-9-D-4169, ICD-9-D-4900:ICD-9-D-5059, ICD-9-D-5064, ICD-9-D-5081, ICD-9-D-5088	ICD-10-D-I2781:ICD-10-D-I279, ICD-10-D-J400:ICD-10-D-J479, ICD-10-D-J600:ICD-10-D-J679, ICD-10-D-J684, ICD-10-D-J701, ICD-10-D-J703		
Coagulopathy	ICD-9-D-2860:ICD-9-D-2871, ICD-9-D-2873:ICD-9-D-2875	ICD-10-D-D65:ICD-10-D-D689, ICD-10-D-D691, ICD-10-D-D693:ICD-10-D-D696		

eTable1 Cont'd.

Congestive Heart Failure	ICD-9-D-39891, ICD-9-D-4280, ICD-9-D-4281, ICD-9-D-42820, ICD-9-D-42821, ICD-9-D-42822, ICD-9-D-42823, ICD-9-D-42830, ICD-9-D-42831, ICD-9-D-42832, ICD-9-D-42833, ICD-9-D-42840, ICD-9-D-42841, ICD-9-D-42842, ICD-9-D-42843, ICD-9-D-4289	ICD-10-D-I150:ICD-10-D-I159		
Coronary Artery Disease	ICD-9-D-4110:ICD-9-D-4149	ICD-10-D-I25:ICD-10-D-I259		
Deficiency Anemia	ICD-9-D-2801:ICD-9-D-2819	ICD-10-D-D508, ICD-10-D-D509, ICD-10-D-D510:ICD-10-D-D539		
Depression	ICD-9-D-2962:ICD-9-D-29636, ICD-9-D-29651:ICD-9-D-29659, ICD-9-D-3004, ICD-9-D-3090:ICD-9-D-3099, ICD-9-D-311	ICD-10-D-F204, ICD-10-D-F3130:ICD-10-D-F315, ICD-10-D-F320:ICD-10-D-F339, ICD-10-D-F341, ICD-10-D-F412, ICD-10-D-F4320:ICD-10-D-F4329		
Diabetes	ICD-9-D-24900:ICD-9-D-25099, ICD-9-D-7902, ICD-9-D-79021, ICD-9-D-79022, ICD-9-D-79029, ICD-9-D-7915, ICD-9-D-7916	ICD-10-D-E080:ICD-10-D-E139		
Diabetes Complicated	ICD-9-D-2504:ICD-9-D-25099	ICD-10-D-E102:ICD-10-D-E108, ICD-10-D-E112:ICD-10-D-E118, ICD-10-D-E142:ICD-10-D-E148, ICD-10-D-E122:ICD-10-D-E128, ICD-10-D-E132:ICD-10-D-E138		
Diabetes Uncomplicated	ICD-9-D-2500:ICD-9-D-25039	ICD-10-D-E100:ICD-10-D-E1011, ICD-10-D-E109, ICD-10-D-E110:ICD-10-D-E1111, ICD-10-D-E119, ICD-10-D-E120, ICD-10-D-E121, ICD-10-D-E129, ICD-10-D-E130:ICD-10-D-E1311, ICD-10-D-E139, ICD-10-D-E140, ICD-10-D-E141, ICD-10-D-E149		
Drug Abuse	ICD-9-D-2920:ICD-9-D-2929, ICD-9-D-3040:ICD-9-D-3049, ICD-9-D-3052:ICD-9-D-3059, ICD-9-D-V6542	ICD-10-D-F110:ICD-10-D-F1699, ICD-10-D-F180:ICD-10-D-F1999, ICD-10-D-Z7151, ICD-10-D-Z7152, ICD-10-D-Z722		
Fluid and Electrolyte Disorder	ICD-9-D-2536, ICD-9-D-2760:ICD-9-D-2769	ICD-10-D-E222, ICD-10-D-E860:ICD-10-D-E878		
HIV	ICD-9-D-042:ICD-9-D-04499	ICD-10-D-B20:ICD-10-D-B2299, ICD-10-D-B24:ICD-10-D-B2499		
Hypertension	ICD-9-D-4010:ICD-9-D-4059	ICD-10-D-I10:ICD-10-D-I159		
Hypothyroidism	ICD-9-D-2409, ICD-9-D-2430:ICD-9-D-2449, ICD-9-D-2461, ICD-9-D-2468	ICD-10-D-E000:ICD-10-D-E039, ICD-10-D-E890		
Ischemic Heart Disease	ICD-9-D-41000:ICD-9-D-41499	ICD-10-D-I21:ICD-10-D-I229		
Liver Disease	ICD-9-D-07022, ICD-9-D-07023, ICD-9-D-07032, ICD-9-D-07033, ICD-9-D-07044, ICD-9-D-07054, ICD-9-D-0706, ICD-9-D-0709, ICD-9-D-4560:ICD-9-D-4562, ICD-9-D-5700:ICD-9-D-5719, ICD-9-D-5722:ICD-9-D-5728, ICD-9-D-5733, ICD-9-D-5734, ICD-9-D-5738, ICD-9-D-5739, ICD-9-D-V427, ICD-9-D-07022, ICD-9-D-07023, ICD-9-D-07032, ICD-9-D-07033ICD-9-D-07044, ICD-9-D-07054, ICD-9-D-0706, ICD-9-D-0709, ICD-9-D-570:ICD-9-D-57099, ICD-9-D-571:ICD-9-D-57199, ICD-9-D-5733, ICD-9-D-5734, ICD-9-D-5738, ICD-9-D-5739, ICD-9-D-V427, ICD-9-D-4560:ICD-9-D-4562, ICD-9-D-5722:ICD-9-D-5728	ICD-10-D-B180:ICD-10-D-B189, ICD-10-D-I850:ICD-10-D-I859, ICD-10-D-I864, ICD-10-D-I982, ICD-10-D-K700:ICD-10-D-K709, ICD-10-D-K7110:ICD-10-D-K7111, ICD-10-D-K713:ICD-10-D-K7151, ICD-10-D-K717, ICD-10-D-K7200:ICD-10-D-K7469, ICD-10-D-K760, ICD-10-D-K762:ICD-10-D-K769Z944, ICD-10-D-B18:ICD-10-D-B1899, ICD-10-D-K700:ICD-10-D-K703, ICD-10-D-K709, ICD-10-D-K713:ICD-10-D-K715, ICD-10-D-K717, ICD-10-D-K73:ICD-10-D-K7399, ICD-10-D-K74:ICD-10-D-K7499, ICD-10-D-K760, ICD-10-D-K762:ICD-10-D-K764, ICD-10-D-K768, ICD-10-D-K769, ICD-10-D-Z944, ICD-10-D-I850, ICD-10-D-I859, ICD-10-D-I864, ICD-10-D-I982, ICD-10-D-K704, ICD-10-D-K711, ICD-10-D-K721, ICD-10-D-K729, ICD-10-D-K765, ICD-10-D-K766, ICD-10-D-K767		

eTable1 Cont'd.

Lymphoma	ICD-9-D-2000:ICD-9-D-20382, ICD-9-D-2386	ICD-10-D-C810:ICD-10-D-C859, ICD-10-D-C880:ICD-10-D-C889, ICD-10-D-C960:ICD-10-D-C969, ICD-10-D-C900:ICD-10-D-C902, ICD-10-D-C9020:ICD-10-D-C9022		
Metastastic Cancer	ICD-9-D-1960:ICD-9-D-1999	ICD-10-D-C770:ICD-10-D-C809		
Multiple Sclerosis	ICD-9-D-340	ICD-10-D-G35		
Non-Alzheimer's Dementia	ICD-9-D-2900, ICD-9-D-29010, ICD-9-D-29011, ICD-9-D-29012, ICD-9-D-29013, ICD-9-D-29020, ICD-9-D-29021, ICD-9-D-2903, ICD-9-D-29040, ICD-9-D-29041, ICD-9-D-29042, ICD-9-D-29043, ICD-9-D-29410, ICD-9-D-29411, ICD-9-D-29420, ICD-9-D-29421, ICD-9-D-33119, ICD-9-D-33182	ICD-10-D-F0150, ICD-10-D-F0151, ICD-10-D-F0280, ICD-10-D-F0281, ICD-10-D-F0390, ICD-10-D-F0391, ICD-10-D-G3109, ICD-10-D-G3183		
Obesity	ICD-9-D-2780, ICD-9-D-27800, ICD-9-D-27801, ICD-9-D-27802, ICD-9-D-27803	ICD-10-D-E660:ICD-10-D-E669		
Orchiectomy				CPT-54520, CPT-54522, CPT-54530, CPT-54535, CPT-54690
Osteoarthritis	ICD-9-D-71500:ICD-9-D-71599	ICD-10-D-M1911:ICD-10-D-M1993		
Other Neurological Disorders	ICD-9-D-3319, ICD-9-D-3320, ICD-9-D-3321, ICD-9-D-3334, ICD-9-D-3335, ICD-9-D-33392, ICD-9-D-3340:ICD-9-D-3359, ICD-9-D-3362, ICD-9-D-3400:ICD-9-D-3419, ICD-9-D-3450:ICD-9-D-3459, ICD-9-D-3481, ICD-9-D-3483, ICD-9-D-7803, ICD-9-D-7843	ICD-10-D-G100:ICD-10-D-G139, ICD-10-D-G200:ICD-10-D-G229, ICD-10-D-G254, ICD-10-D-G255, ICD-10-D-G312, ICD-10-D-G318, ICD-10-D-G319, ICD-10-D-G320:ICD-10-D-G329, ICD-10-D-G350:ICD-10-D-G379, ICD-10-D-G400:ICD-10-D-G419, ICD-10-D-G931, ICD-10-D-G934, ICD-10-D-R470, ICD-10-D-R560:ICD-10-D-R569		
Peripheral Vascular Disease	ICD-9-D-093, ICD-9-D-4373, ICD-9-D-440:ICD-9-D-44099, ICD-9-D-441:ICD-9-D-44199, ICD-9-D-4431:ICD-9-D-4439, ICD-9-D-4471, ICD-9-D-5571, ICD-9-D-5579, ICD-9-D-V434	ICD-10-D-I70:ICD-10-D-I7099, ICD-10-D-I71:ICD-10-D-I7199, ICD-10-D-I731, ICD-10-D-I738, ICD-10-D-I739, ICD-10-D-I771, ICD-10-D-I790, ICD-10-D-I792, ICD-10-D-K551, ICD-10-D-K558, ICD-10-D-K559, ICD-10-Z958, ICD-10-D-Z959		
Paralysis	ICD-9-D-3341, ICD-9-D-342:ICD-9-D-34299, ICD-9-D-343:ICD-9-D-34399, ICD-9-D-3440:ICD-9-D-3449	ICD-10-D-G041, ICD-10-D-G114, ICD-10-D-G801, ICD-10-D-G802, ICD-10-D-G81:ICD-10-D-G8199, ICD-10-D-G82:ICD-10-D-G8299, ICD-10-D-G830:ICD-10-D-G834, ICD-10-D-G839		
Parkinson's Disease	ICD-9-D-332, ICD-9-D-3320	ICD-10-D-G20, ICD-10-D-G214		
Peptic Ulcer Disease	ICD-9-D-531:ICD-9-D-53499, ICD-9-D-53170, ICD-9-D-53171, ICD-9-D-53190, ICD-9-D-53191, ICD-9-D-5327, ICD-9-D-5329, ICD-9-D-5337, ICD-9-D-5339, ICD-9-D-5347, ICD-9-D-5349	ICD-10-D-K25:ICD-10-D-K2899, ICD-10-D-K257, ICD-10-D-K259, ICD-10-D-K267, ICD-10-D-K269, ICD-10-D-K277, ICD-10-D-K279, ICD-10-D-K287, ICD-10-D-K289		

eTable1 Cont'd.

Prostate Cancer	ICD-9-D-185, ICD-9-D-2222, ICD-9-D-2334, ICD-9-D-2365, ICD-9-D-60010, ICD-9-D-60011, ICD-9-D-V1046, ICD-9-D-V7644, ICD-9-D-60000, ICD-9-D-60001, ICD-9-D-60020, ICD-9-D-60021, ICD-9-D-6003, ICD-9-D-60090, ICD-9-D-60091, ICD-9-D-6012, ICD-9-D-6018, ICD-9-D-6020, ICD-9-D-6021, ICD-9-D-6022, ICD-9-D-6023, ICD-9-D-6028, ICD-9-D-6029, ICD-9-D-79093	ICD-10-D-C61, ICD-10-D-D075, ICD-10-D-D291, ICD-10-D-D400, ICD-10-D-N402, ICD-10-D-N403, ICD-10-D-N412, ICD-10-D-N418, ICD-10-D-N419, ICD-10-D-N420, ICD-10-D-N421, ICD-10-D-N423, ICD-10-D-N4230, ICD-10-D-N4232, ICD-10-D-N4239, ICD-10-D-N4283, ICD-10-D-N4289, ICD-10-D-N429, ICD-10-D-Q554, ICD-10-D-R972, ICD-10-D-R9720, ICD-10-D-R9721, ICD-10-D-Z125		
Prostatectomy				CPT-55801, CPT-55810, CPT-55812, CPT-55815, CPT-
Psychoses	ICD-9-D-29381:ICD-9-D-293.89, ICD-9-D-2950:ICD-9-D-29599, ICD-9-D-29604, ICD-9-D-29614, ICD-9-D-29644, ICD-9-D-29654, ICD-9-D-2970:ICD-9-D-2989	ICD-10-D-F200:ICD-10-D-F209, ICD-10-D-F22:ICD-10-D-F259, ICD-10-D-F28, ICD-10-D-F29, ICD-10-D-F302, ICD-10-D-F312, ICD-10-D-F315		
Pulmonary Heart Disease	ICD-9-D-4150:ICD-9-D-41799	ICD-10-D-I26:ICD-10-D-I279		
Renal Disease	ICD-9-D-40301, ICD-9-D-40311, ICD-9-D-40391, ICD-9-D-40402, ICD-9-D-40403, ICD-9-D-40412, ICD-9-D-40413, ICD-9-D-40492, ICD-9-D-40493, ICD-9-D-582:ICD-9-D-58299, ICD-9-D-5830:ICD-9-D-5837, ICD-9-D-585:ICD-9-D-58599, ICD-9-D-586:ICD-9-D-58699, ICD-9-D-5880, ICD-9-D-V420, ICD-9-D-V451, ICD-9-D-V56:ICD-9-D-V5699	ICD-10-D-I120, ICD-10-D-I131, ICD-10-D-N032:ICD-10-D-N037, ICD-10-D-N052:ICD-10-D-N057, ICD-10-D-N18:ICD-10-D-N1899, ICD-10-D-N19:ICD-10-D-N1999, ICD-10-D-N250, ICD-10-D-Z490:ICD-10-D-Z492, ICD-10-D-Z940, ICD-10-D-Z992		
Renal Failure	ICD-9-D-40301, ICD-9-D-40311, ICD-9-D-40391, ICD-9-D-40402, ICD-9-D-40403, ICD-9-D-40412, ICD-9-D-40413, ICD-9-D-40492, ICD-9-D-40493, ICD-9-D-5850:ICD-9-D-5869, ICD-9-D-5880, ICD-9-D-V420, ICD-9-D-V451, ICD-9-D-V560:ICD-9-D-V569	ICD-10-D-I120, ICD-10-D-I1311, ICD-10-D-N180:ICD-10-D- NI9, ICD-10-D-N250, ICD-10-D-Z4901:ICD-10-D-Z4902, ICD-10-D-Z940, ICD-10-D-Z992		
Rheumatoid Arthritis	ICD-9-D-7140, ICD-9-D-7142	ICD-10-D-M0520:ICD-10-D-M061		
Stroke	ICD-9-D-430, ICD-9-D-431, ICD-9-D-432, ICD-9-D-4320, ICD-9-D-4321, ICD-9-D-4329, ICD-9-D-43300, ICD-9-D-4331, ICD-9-D-43310, ICD-9-D-43311, ICD-9-D-43320, ICD-9-D-43321, ICD-9-D-43330, ICD-9-D-43331, ICD-9-D-43381, ICD-9-D-43390, ICD-9-D-43391, ICD-9-D-43400, ICD-9-D-43401, ICD-9-D-43410, ICD-9-D-43411, ICD-9-D-43490, ICD-9-D-43491	ICD-10-D-I6300, ICD-10-D-I63011, ICD-10-D-I63012, ICD-10-D-I63031, ICD-10-D-I63032, ICD-10-D-I6310, ICD-10-D-I63139, ICD-10-D-I63232, ICD-10-D-I63233, ICD-10-D-I63239, ICD-10-D-I63312, ICD-10-D-I6339, ICD-10-D-I6340, ICD-10-D-I63411, ICD-10-D-I63432, ICD-10-D-I6350, ICD-10-D-I63512, ICD-10-D-I63519, ICD-10-D-I63529, ICD-10-D-I6359, ICD-10-D-I638, ICD-10-D-I639		
Tobacco Use	ICD-9-D-3051, ICD-9-D-98984, ICD-9-D-V1582	ICD-10-D-F17220, ICD-10-D-F17221, ICD-10-D-F17223, ICD-10-D-F17228, ICD-10-D-F17229, ICD-10-D-F17290, ICD-10-D-F17291, ICD-10-D-F17293, ICD-10-D-F17298, ICD-10-D-F17299, ICD-10-D-Z720		
Valvular Disease	ICD-9-D-0932, ICD-9-D-3940:ICD-9-D-3979, ICD-9-D-4240:ICD-9-D-4249, ICD-9-D-7463:ICD-9-D-7466, ICD-9-D-V422, ICD-9-D-V433	ICD-10-D-A5200:ICD-10-D-A5209, ICD-10-D-I050:ICD-10-D-I089, ICD-10-D-I091, ICD-10-D-I098, ICD-10-D-I340:ICD-10-D-I39, ICD-10-D-Q23O:ICD-10-D-Q233, ICD-10-D-Z952, ICD-10-D-Z954		

eTable1 Cont'd.

Weight Loss	ICD-9-D-2600:ICD-9-D-2639, ICD-9-D-78321:ICD-9-D-78322, ICD-9-D-7994	ICD-10-D-E400:ICD-10-D-E46, ICD-10-D-R634, ICD-10-D-R64		
Abiraterone			GENERIC_DRUG-ABIRATERONE_ACETATE, DRUG-ZYTIGA	
Bicalutamide			GENERIC_DRUG-BICALUTAMIDE, DRUG-CASODEX	
Degarelix			GENERIC_DRUG-DEGARELIX_ACETATE, DRUG-FIRMAGON	
Enzalutamide			GENERIC_DRUG-ENZALUTAMIDE	
Flutamide			GENERIC_DRUG-FLUTAMIDE	
Goserelin			GENERIC_DRUG-GOSERELIN_ACETATE, DRUG-ZOLADEX	
Histrelin			GENERIC_DRUG-HISTRELIN_ACETATE, DRUG-VANTAS	
Ketoconazole			GENERIC_DRUG-KETOCONAZOLE, DRUG-NIZORAL	
Leuprolide			GENERIC_DRUG-LEUPROLIDE/NORETHINDRONE_ACET, GENERIC_DRUG-LEUPROLIDE_ACETATE, DRUG-LEUPROLIDE_ACETATE DRUG-ELIGARD DRUG-LUPRON DRUG-LUPRON_DEPOT DRUG-LUPRON_DEPOT-PED	
Nilutamide			GENERIC_DRUG-NILUTAMIDE, DRUG-NILANDRON	
Triptorelin			GENERIC_DRUG-TRIPTORELIN_PAMOATE, DRUG-TRELSTAR	

eFigure1: Relative Risk of Propensity Score-matched Patients with Exposure to ATT to Develop NDD

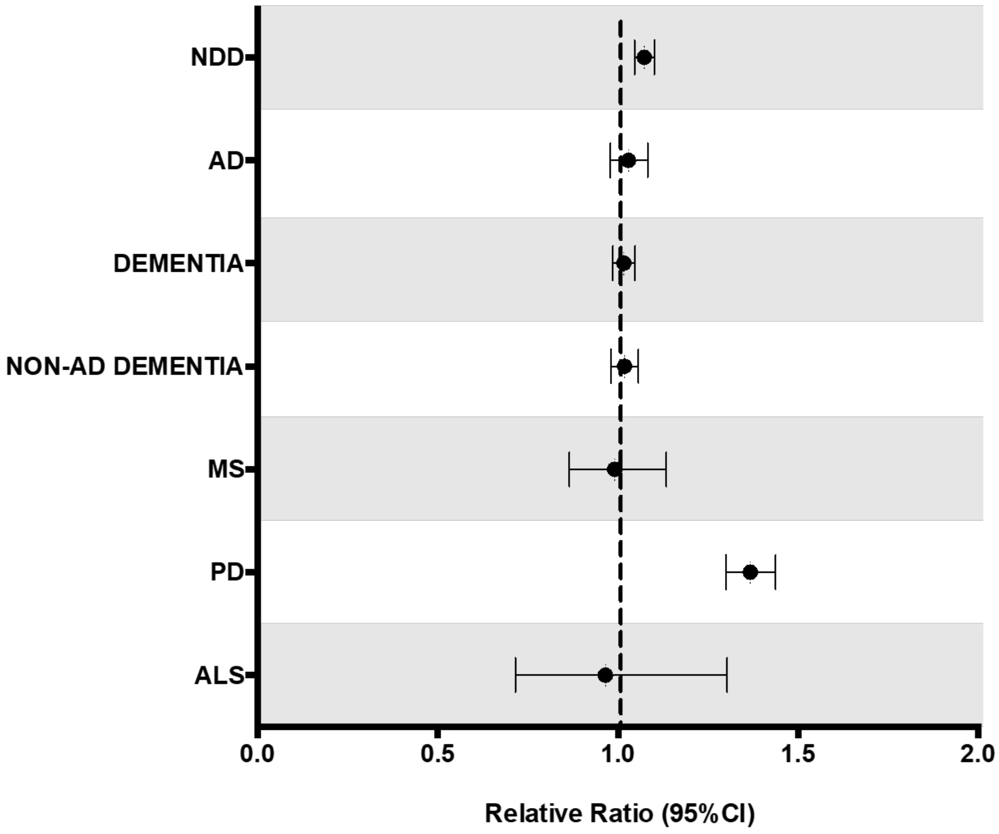

RR (95CI)	P-value
1.07 (1.05-1.10)	<.001
1.03 (0.98-1.08)	.26
1.02 (0.99-1.05)	.31
1.02 (0.98-1.06)	.37
0.99 (0.87-1.13)	.92
1.37 (1.30-1.44)	<.001
0.97 (0.71-1.30)	.88

eTable 2. Summary of means and balance improvement for propensity score matching criteria

	All Data			Matched Data			% of Balance Imoprovement
	Means Treated	Means Control	Std. Mean Diff.	Means Treated	Means Control	Std. Mean Diff.	Std. Mean Diff.
distance	0.138	0.125	0.290	0.138	0.138	0.000	99.997
COMORB.AcuteMyocardialInfarctionTRUE	0.131	0.125	0.018	0.131	0.125	0.017	8.182
COMORB.AlcoholAbuseTRUE	0.058	0.060	-0.010	0.058	0.056	0.010	-5.236
COMORB.AsthmaTRUE	0.079	0.069	0.038	0.079	0.076	0.012	69.400
COMORB.BloodLossAnemiaTRUE	0.042	0.037	0.024	0.042	0.039	0.017	29.149
COMORB.COPDTRUE	0.291	0.276	0.032	0.291	0.288	0.006	81.537
COMORB.CancerTRUE	0.478	0.369	0.218	0.478	0.480	-0.004	97.940
COMORB.CardiacArrhythmiasTRUE	0.321	0.295	0.055	0.321	0.316	0.009	82.746
COMORB.CerebrovascularDiseaseTRUE	0.306	0.275	0.067	0.306	0.304	0.005	92.090
COMORB.ChronicKidneyDiseaseTRUE	0.228	0.201	0.066	0.228	0.219	0.023	64.490
COMORB.ChronicPulmonaryDiseaseTRUE	0.336	0.316	0.042	0.336	0.330	0.013	69.965
COMORB.CoagulopathyTRUE	0.120	0.106	0.043	0.120	0.116	0.014	66.799
COMORB.CongestiveHeartFailureTRUE	0.116	0.108	0.025	0.116	0.111	0.018	30.748
COMORB.CoronaryArteryDiseaseTRUE	0.423	0.388	0.070	0.423	0.416	0.013	81.365
COMORB.DeficiencyAnemiaTRUE	0.172	0.152	0.052	0.172	0.167	0.014	72.435
COMORB.DepressionTRUE	0.254	0.238	0.036	0.254	0.248	0.013	63.748
COMORB.DiabetesComplicatedTRUE	0.247	0.208	0.090	0.247	0.239	0.018	80.079
COMORB.DiabetesUncomplicatedTRUE	0.403	0.364	0.081	0.403	0.397	0.013	84.044
COMORB.DrugAbuseTRUE	0.050	0.045	0.023	0.050	0.046	0.017	26.853
COMORB.FluidAndElectrolyteDisordersTRUE	0.298	0.278	0.043	0.298	0.291	0.015	63.942
COMORB.HIVTRUE	0.008	0.006	0.032	0.008	0.008	0.007	76.699
COMORB.IschemicHeartDiseaseTRUE	0.364	0.334	0.062	0.364	0.359	0.010	83.536
COMORB.LymphomaTRUE	0.036	0.033	0.012	0.036	0.034	0.008	33.493
COMORB.MetastaticCancerTRUE	0.117	0.059	0.179	0.117	0.120	-0.009	94.967
COMORB.ObesityTRUE	0.287	0.261	0.059	0.287	0.279	0.018	68.930
COMORB.OsteoarthritisTRUE	0.416	0.378	0.078	0.416	0.411	0.011	86.236
COMORB.OtherNeurologicalDisordersTRUE	0.106	0.094	0.039	0.106	0.103	0.008	79.216
COMORB.PVDTRUE	0.310	0.269	0.089	0.310	0.305	0.012	86.994
COMORB.PsychosesTRUE	0.038	0.037	0.004	0.038	0.036	0.011	-162.655
COMORB.RenalFailureTRUE	0.160	0.144	0.045	0.160	0.154	0.017	63.292
COMORB.RheumatoidArthritisTRUE	0.031	0.026	0.028	0.031	0.030	0.010	65.623
COMORB.SevereLiverDiseaseTRUE	0.008	0.008	0.002	0.008	0.007	0.009	-515.419
COMORB.SolidTumorWithoutMetastasisTRUE	0.456	0.345	0.224	0.456	0.460	-0.008	96.551
COMORB.TobaccoUseTRUE	0.242	0.254	-0.029	0.242	0.237	0.012	58.518
COMORB.ValvularDiseaseTRUE	0.249	0.224	0.059	0.249	0.246	0.008	86.727
COMORB.WeightLossTRUE	0.131	0.129	0.007	0.131	0.127	0.012	-67.304
COMP.AcuteKidneyInjuryTRUE	0.161	0.144	0.046	0.161	0.152	0.025	44.723
COMP.CapsulitisTRUE	0.028	0.024	0.023	0.028	0.026	0.011	54.505
COMP.CardiacArrestTRUE	0.013	0.014	-0.006	0.013	0.013	0.007	-28.380

eFigure2: Kaplan-Meier Survival Curves for Patients with or without Exposure to ATT to Develop NDD

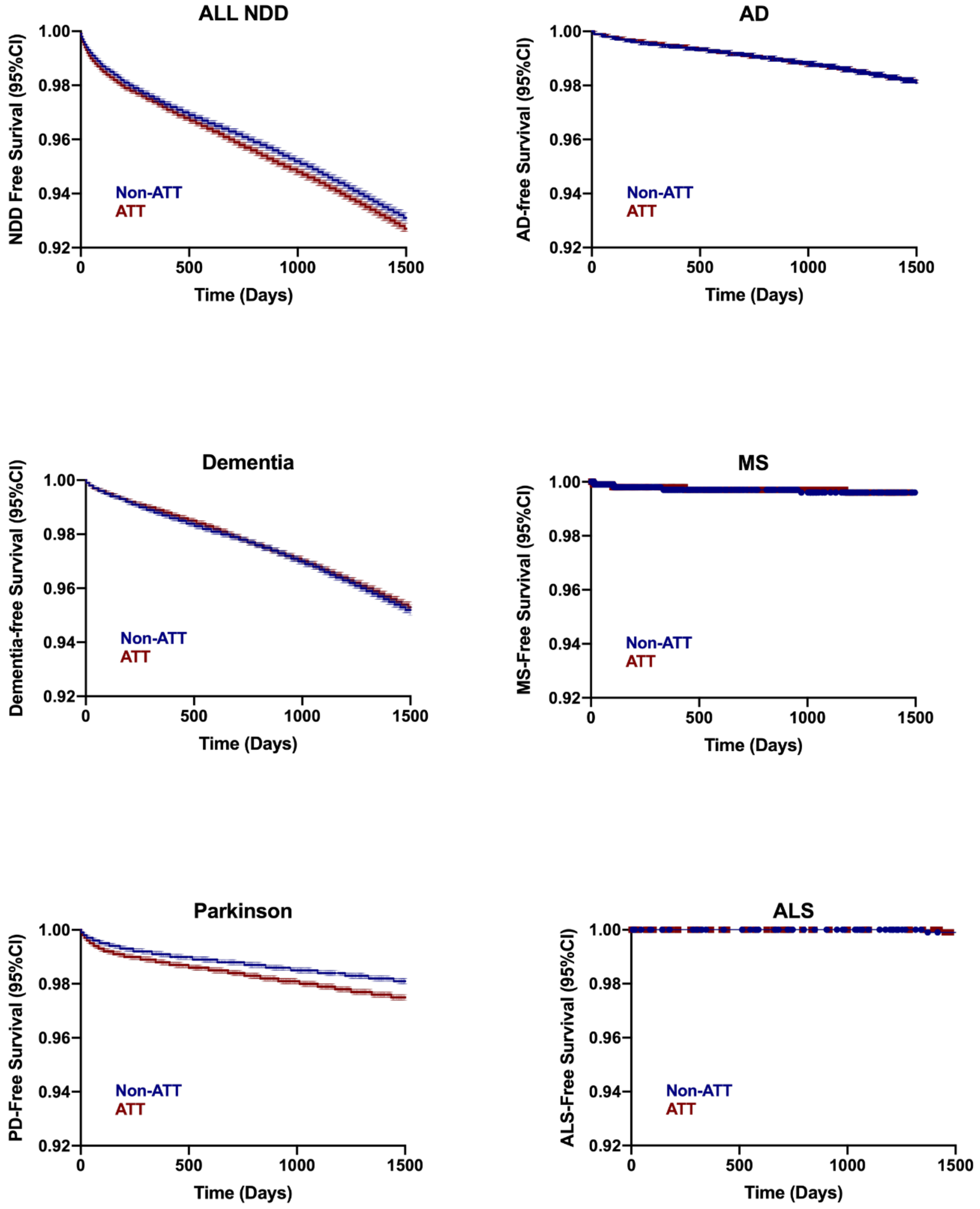

eFigure3: Drug-Target Interaction Network for Each ATT Drug

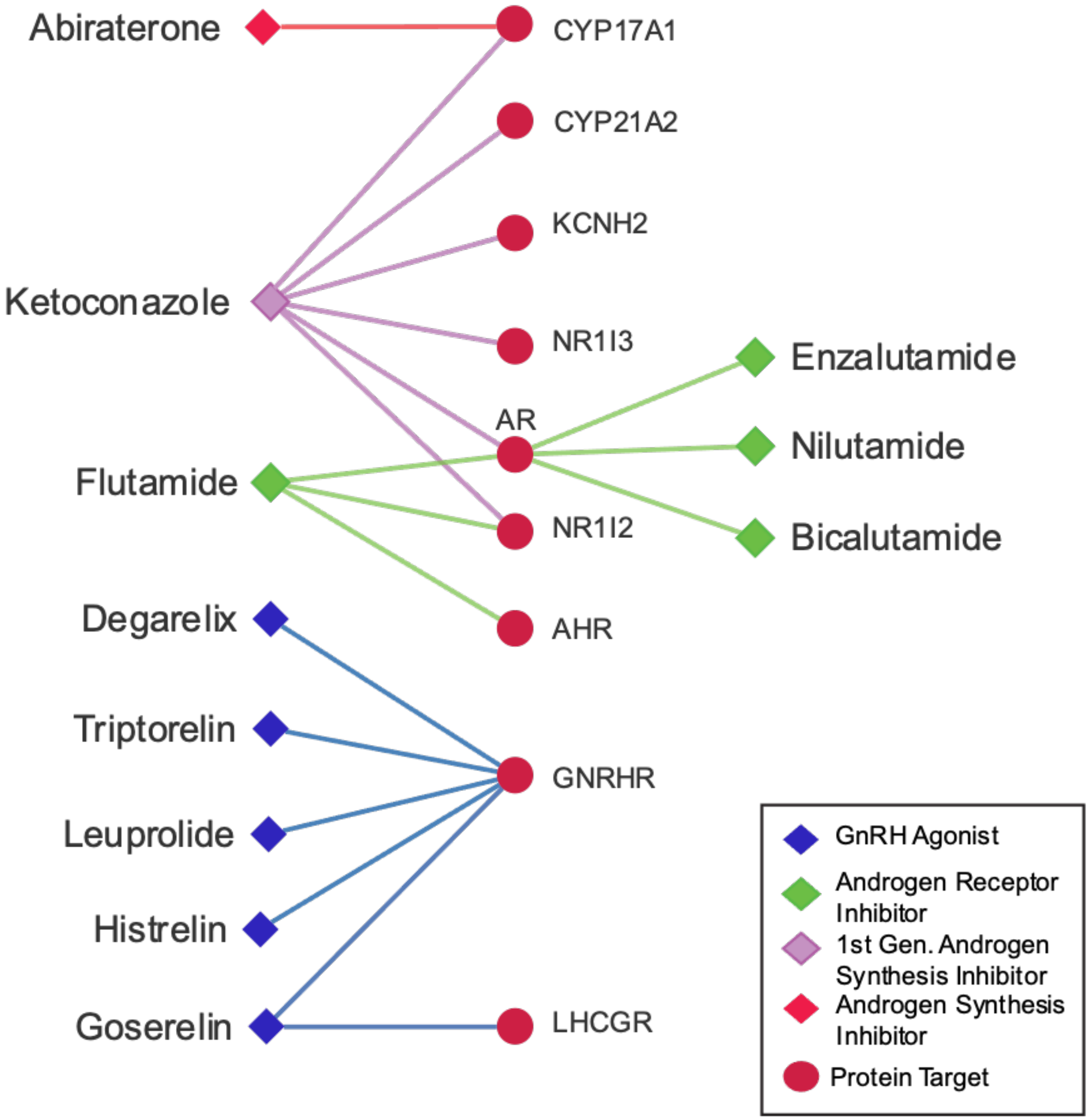

eFigure4: Patient Breakdown and Study Design of Patients Who Did Not Undergo Surgical Procedures (Prostatectomy or Orchiectomy)

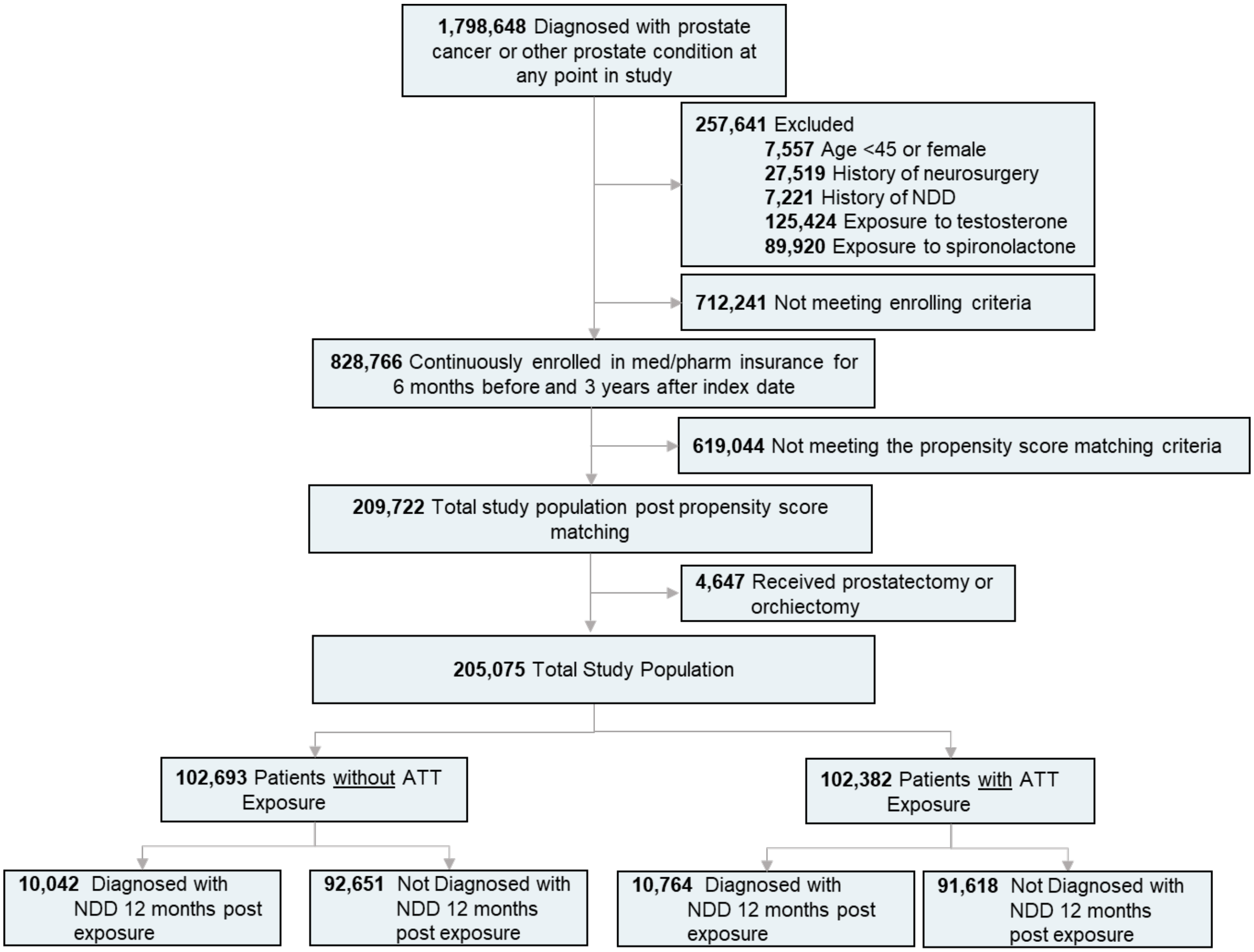

eTable3: Relative Risk of Patients With Exposure to ATT Who Did or Did Not Undergo Surgical Procedures to Develop NDD

**ORCHIECTOMY**		
NDD		
Impact of	ATT in Patients Without Surgery	ATT + Surgery vs No Intervention
RR	1.48	2.25
95% CI	1.44-1.53	1.65-3.03
p-value	<.001	<.001
NNT	35.52	13.75

**PROSTATECTOMY**		
NDD		
Impact of	ATT in Patients Without Surgery	ATT + Surgery vs No Intervention
RR	1.48	0.8
95% CI	1.44-1.53	0.67-0.97
p-value	<.001	0.02
NNT	35.22	86.81

eTable4: A) Effect of Prednisone Exposure on Incidence of NDD. B) Number of Patients With Exposure to Abiraterone Who are Also Exposed to Prednisone

A)

	NDD	NO NDD	Total
**PREDNISONE**	195	2517	2712
**CONTROL**	16	265	281

B)

**Abiraterone**	2993	
+ prednisone	2712	90.61%
- prednisone	281	9.39%
